# It’s not in my head: a qualitative analysis of experiences of discrimination in people with mental health and substance use conditions seeking physical healthcare

**DOI:** 10.3389/fpsyt.2023.1285431

**Published:** 2023-10-16

**Authors:** Ruth Cunningham, Fiona Imlach, Tracy Haitana, Susanna Every-Palmer, Cameron Lacey, Helen Lockett, Debbie Peterson

**Affiliations:** ^1^Department of Public Health, University of Otago Wellington, Wellington, New Zealand; ^2^Māori/Indigenous Health Institute (MIHI), University of Otago Christchurch, Christchurch, New Zealand; ^3^Department of Psychological Medicine, University of Otago Wellington, Wellington, New Zealand

**Keywords:** discrimination, health care, mental health, quality of care, overshadowing, severe mental disorder, substance use disorder

## Abstract

**Introduction:**

Clinician bias contributes to lower quality healthcare and poorer health outcomes in people with mental health and substance use conditions (MHSUC). Discrimination can lead to physical conditions being overlooked (diagnostic overshadowing) or substandard treatment being offered to people with MHSUC. This research aimed to utilise experiences of people with MHSUC to identify discrimination by clinicians, including the role of clinician’s beliefs and assumptions in physical health service provision.

**Methods:**

We surveyed people with MHSUC who accessed physical healthcare services. Of 354 eligible participants, 253 responded to open-ended questions about experiences of those services. Thematic descriptive analysis of survey responses was completed using existing stigma frameworks and inductive coding.

**Results:**

One dominant theme from survey responses was that diagnostic overshadowing by clinicians was driven by clinician mistrust. Another theme was that clinicians assumed respondent’s physical symptoms, including pain, were caused by MHSUC. This influenced decisions not to initiate investigations or treatment. Respondents perceived that clinicians focused on mental health over physical health, contributing to suboptimal care.

**Discussion:**

Discrimination based on MHSUC leads to poor quality care. Health systems and clinicians need to focus quality improvement processes on access to and delivery of equitable physical healthcare to people with MHSUC, address stereotypes about people with MHSUC and improve integration of mental and physical healthcare.

## 1. Introduction

People with mental health and substance use conditions (MHSUC) experience worse outcomes from physical health conditions than those without MHSUC (1–4). This can be attributed in part to poorer quality physical healthcare, including lower rates of timely, appropriate diagnosis and treatment (5, 6).

People with MHSUC presenting to a health service with a physical complaint commonly experience the complaint being dismissed or ignored (7–10). When physical symptoms are misattributed to MHSUC, with subsequent missed or incorrect diagnoses, this is described as diagnostic overshadowing (11, 12).

Even when physical conditions are recognized, differential outcomes may still occur if clinicians provide different treatments or fail to address barriers to care for people with MHSUC (11). Therapeutic pessimism or overshadowing can contribute to clinicians developing inferior treatment plans for people with MHSUC, driven by negative beliefs about a person’s capacity and ability to comply or respond to treatment (11, 13).

Bias from clinicians plays an important role in both diagnostic and therapeutic overshadowing (11, 14, 15). Bias encompasses a range of factors, from lack of experience or knowledge about mental health (ignorance), negative beliefs about people with MHSUC (stereotypes), negative attitudes and emotions towards people with MHSUC (prejudice) and discriminatory behavior (discrimination) (16, 17). Bias can also be unconscious or implicit, and bias against people with MHSUC occurs in clinicians at similar high levels to the general population (18, 19).

Previous research in Aotearoa New Zealand (NZ) reported that 23% of people with MHSUC experienced discrimination from health services (20). Examples of discrimination include acting on stereotypes and prejudice, such as clinicians avoiding patients with MHSUC due to unwarranted fear of violence or discomfort with mental illness (21) and patients with MHSUC being denied weight loss surgery due to unsubstantiated assumptions they will have poorer outcomes (22). Discrimination from clinicians can also deter people from seeking treatment (23), creating further barriers to appropriate diagnosis and treatment of physical conditions.

Although qualitative research has examined reasons for overshadowing from the perspective of clinicians (10, 21, 24), exploring discrimination from the perspective of people with MHSUC is less common and usually limited to describing discriminatory behavior without identifying underlying stereotypes (25–28). We surveyed people with MHSUC in NZ who had accessed healthcare for a physical health condition, drawing on methodology that also used patient perspectives to critique service quality issues (29, 30).

The aims of the research were:

- To describe how people with MHSUC experienced discrimination in physical health services, including diagnostic and therapeutic overshadowing.
- To use the observations and reports of people with MHSUC to explore likely underlying beliefs of clinicians that lead to discrimination in physical healthcare.

## 2. Materials and methods

Experiences of physical healthcare in people with MHSUC were collected through an anonymous online Qualtrics survey that included both closed and open-ended questions and ran from 31 January to 1 April 2022.

### 2.1. Recruitment and sample

People were recruited through snowballing methods using digital media, starting with social media outreach through Facebook and Twitter by the researcher team and research advisory group and distribution through online newsletters and email lists from other organizations (e.g., Government and non-governmental organizations and services, Māori health networks and providers).

The survey site was accessed 488 times. Four-hundred-and-eight people agreed to participate, and 354 eligible individuals were included in the final dataset. Eligibility criteria were:

- Use of primary or secondary healthcare services for MHSUC in the past 5 years
- Engagement with any health care service for a physical health issue in the past 5 years
- Age 18 or over.

People who did not answer any questions about physical healthcare services were excluded, as was one duplicate response. The analysis sample for this paper included only those who answered at least one of the open-ended questions (*n* = 253).

### 2.2. Survey content

The survey was divided into four sections covering mental health and addiction service use, physical health service use (across five types of services), stigma and discrimination, and demographics. Demographic questions included age, gender, ethnicity, sexual orientation, and MHSUC diagnoses. The survey questionnaire was reviewed by a research advisory group, which included clinicians and people with lived experience of MHSUC. The responses to ten open-ended questions formed the basis of the qualitative analysis (Table 1).

**Table 1:** Open-ended questions for analysis.

Open-ended questions
Please tell us more about your experiences of physical healthcare from general practice (GP) services
Please tell us more about your experiences of physical healthcare from emergency departments
Please tell us more about your experiences of physical healthcare in hospital services
Please tell us more about your experiences of physical healthcare from chemists or pharmacies
Please tell us more about your experiences of physical healthcare from other health services (e.g., kaupapa Māori health service, physiotherapy, dietetic service, naturopath service)
Please tell us more about being accompanied by a mental health or addiction staff member to an appointment for your physical health
Please tell us more about deciding not to seek help and/or continue with treatment for a physical health problem, in case you were treated unfairly due to your experience of mental health or addiction
Please tell us more about about being treated unfairly due to your ethnicity, age, gender, sexual orientation or disability when seeking help for a physical health problem
How do you think physical health care could be improved for people with mental illness or addiction issues?
Do you have any other comments you wish to make about the topics raised in this survey?

Ethics approval was granted by the Southern Health and Disability Ethics Committee (21/STH/216). Information about the survey, including maintenance of privacy and confidentiality, and contact details for support was provided in the survey introduction. Informed consent was assumed once participants engaged with the online survey. Where identifying information was provided by participants (in order to volunteer for participation in interviews or to receive study results) this information was removed prior to analysis and stored separately and was not accessible to study authors conducting analyzes.

### 2.3. Data analysis

Responses to the open-ended questions were imported into NVivo v1.6.1 (QSR International) in an anonymised form. We used theory-driven thematic analysis (31), starting with deductive coding using pre-existing frameworks on stigma (16, 17) to classify experiences of discrimination. Investigators (FI and DP) independently reviewed survey responses to identify sections of text that described discrimination, prejudice and stereotypes. These sections were analyzed inductively to infer clinicians’ underlying assumptions as perceived and reported by respondents, drawing on the respondents’ observations of clinician demeanor and behavior and their interpretations of these, particularly within the context of delayed, missed or incorrect physical health diagnoses, but also where physical health diagnoses were unknown or unclear. The investigators discussed and developed themes based on respondents’ common experiences as an iterative process with team input and review (Table 2). Quotes to demonstrate themes were inserted verbatim with grammatical corrections to aid clarity and identifiers indicating age range (NA = no age given) and gender (W = woman, M = man).

**Table 2:** Project team expertise and background.

Team member	Expertise/background
CL	Psychiatry, mental health research, Māori health
DP	Mental health research, qualitative research, lived experience
FI	Public health/epidemiology, mixed methods research
HL	Mental health and substance use research, practice and policy
RC	Public health/epidemiology, mental health research
SE-P	Psychiatry, mental health research
TH	Psychology, mental health research, Māori health, qualitative research

## 3. Results

### 3.1. Respondent characteristics

Respondents were predominantly female and compared to the general population were younger and more likely to identify with the rainbow community (Table 3). Depression and anxiety were the most frequently reported MHSUC diagnoses and multiple diagnoses were common.

**Table 3:** Characteristics of respondents.

Characteristic	*n* (%) for total survey sample	*n* (%) for those who responded to open-ended questions
*Age*		
18–25 years old	57 (16%)	43 (17%)
26–35 years old	91 (26%)	65 (26%)
36–45 years old	65 (18%)	49 (19%)
46–54 years old	51 (14%)	42 (17%)
55+ years old	42 (12%)	37 (15%)
Missing	48 (14%)	19 (8%)
*Gender*		
Female	228 (64%)	172 (68%)
Gender diverse	15 (4%)	13 (5%)
Male	59 (17%)	44 (17%)
Prefer not to answer	4 (1%)	4 (2%)
Missing	48 (14%)	20 (8%)
*Ethnicity*		
Māori	58 (16%)	47 (19%)
Non-Māori	245 (69%)	185 (73%)
Missing	51 (14%)	21 (8%)
*Sexual orientation*		
Heterosexual	197 (56%)	149 (59%)
LGBQA+	107 (30%)	83 (33%)
Missing	50 (14%)	21 (8%)
*Diagnosis*^a^		
Addiction	58 (16%)	44 (17%)
Anxiety	225 (64%)	168 (66%)
Bipolar disorder or schizophrenia	59 (17%)	50 (20%)
Depression	241 (68%)	181 (77%)
Personality disorder	41 (12%)	35 (14%)
Post-traumatic stress disorder	54 (15%)	42 (17%)
*Number of diagnoses*		
1	43 (12%)	36 (14%)
2	114 (32%)	85 (34%)
3	79 (22%)	58 (23%)
4+	53 (14%)	44 (17%)
Missing	65 (18%)	30 (12%)
Total	354	253

a This percentage is for all those who reported a diagnosis in the whole sample; the proportions are higher if people who did not report any diagnosis are excluded from the denominator.

### 3.2. Overview of themes

Three themes (clinician’s beliefs) were evident from the descriptive analysis of factors that people with MHSUC considered led to discrimination from clinicians when seeking physical healthcare. Themes included that in people with MHSUC, MHSUC is responsible for physical symptoms (with sub-themes around physical symptoms being psychosomatic, caused by anxiety and pain as an unreal symptom), that people with MHSUC are untrustworthy (particularly those who need controlled drugs or pain management) and that mental and physical healthcare were competing priorities (either mental or physical health takes the focus).

### 3.3. In people with MHSUC, MHSUC is responsible for physical symptoms

A predominant theme from survey respondents centered around the experience of clinicians assuming that their MHSUC was responsible for or explained their physical symptoms. As a result, respondents reported that their physical symptoms were dismissed, leading to delayed investigations, diagnosis and treatment – or no investigation despite an underlying physical cause. Respondents reported that they were not treated in the same way as someone without a history of MHSUC.

I had to argue with a doctor about the cause of dehydration and difficulty swallowing. He put it down to depression and a history of eating disorders. Turned out I had thrush in my mouth and oesophagus after being on antibiotics. (W, 36–45)

Within this theme, sub-themes emerged relating to physical symptoms in people with MHSUC being ascribed as psychosomatic, or due to anxiety and stress, and the physical symptom of pain being not real or caused by MHSUC.

#### 3.3.1. Physical symptoms in people with MHSUC are psychosomatic

Respondents reported that clinicians assumed their physical health symptoms were either psychosomatic or not real. Whether clinicians distinguished between psychosomatic conditions and “feigning” was unclear, but respondents were left with the impression that their symptoms were imagined.

I was in for cardiac issues. When ECGs were done because of chest pain and came back normal it was suggested it could be because of my bipolar by nurses again. It was in fact my Duromine that I was on for weight loss, so it wasn’t in my head at all. (W, 55+)

Although respondents frequently experienced clinicians telling them that physical complaints were psychosomatic or due to their MHSUC, they were not told what this meant or what to do about the symptoms they were experiencing, leaving them unclear how to manage and reluctant to raise the same or other concerns in the future. Respondents experienced this as a bind, where the onus was on them to prove their symptoms were not caused by their MHSUC, and if they could not, they were left without treatment or support.

In some instances, my mental health issues are blamed for my physical health issues but it is never explained why my [mental health] is to blame. I leave feeling hopeless and confused.(W, 26-35)

#### 3.3.2. Physical symptoms in people with MHSUC are due to anxiety and stress

Respondents reported that anxiety and stress were the primary reasons given by clinicians to explain physical symptoms, and the reasons for not investigating further. Examples of this spanned both primary and secondary healthcare settings. Several respondents who were initially told that their physical symptoms were due to anxiety or stress were later diagnosed with significant health conditions, including lupus, a kidney infection, a viral liver infection, endometriosis, brachial neuritis and postural tachycardia syndrome.

My GP often tries to blame any physical problem I have on my anxiety. I know my own anxiety pretty well now, I know what it feels like and how it behaves. It frustrates me when my GP is not willing to investigate my symptoms and just says “it could be your anxiety.” (W, 26–35)

#### 3.3.3. The physical symptom of pain in people with MHSUC is not real or is caused by MHSUC

A special category within this theme related to pain as a physical symptom. People with MHSUC complaining of pain experienced an even stronger sense that their clinicians believed the pain to be imaginary or fabricated and did not warrant treatment. People were given the impression that their MHSUC caused or exacerbated pain, or they could not achieve pain control unless their MHSUC was better managed.

In the last 5 years I have been struggling to get a diagnosis and get treatment for on-going pelvic and back pain as a result of adenomyosis, a pars defect and a few other factors. My mental health would be brought up in every appointment and often blamed for my inability to control my pain levels. [This was] despite already seeing a psychologist and psychiatrist, being on medication, and being in decent control of my mental health. (W, 26–35)

Poor treatment of the symptom of pain could relate to mistrust of people with MHSUC (and reluctance to prescribe pain medication, see below) and/or lack of knowledge about the nature and management of pain. One respondent reported her experience: “I was told that I could not possibly be in pain as my car accident was years ago. The woman told me to repeat after her, ‘tissue damage repairs itself after 3 months so I am not in pain.’” (W, 46–54) That this occurred at a pain clinic suggests that ignorance was not the root cause of this dismissal, as such clinics routinely assess people with pain that persists in the absence of tissue damage.

### 3.4. People with MHSUC are untrustworthy

Respondents recounted how they were not believed when they reported physical health symptoms, with an implicit or sometimes explicit assumption that they were making the symptoms up, exaggerating or even outright lying. The presence of an MHSUC diagnosis was presumed to indicate an unreliable narrator.

I broke my tailbone and sacrum I was called a liar, I'm faking it, etc etc. Turns out was so serious, my insides were prolapsed, tailbone removed, sacrum has 3 pins in but took me 2 years, lots of tears … hundreds of pain meds for them to believe me. (W, 46–54)

Respondents felt ignored, dismissed and not listened to. Respondents wanted their physical health concerns to be taken seriously, independently from their MHSUC diagnosis, and without fear of their symptoms being pre-determined as having a psychological aetiology.

Doctors have been quite dismissive, I've felt, of the physical symptoms I’m experiencing, almost always putting it down to my mental health challenges which at times is frustrating. I just wish they would listen a bit more and not immediately discount physical health complaints because I have a mental health diagnosis. (W, NA)

The experience of being disbelieved and dismissed could have negative consequences for people’s mental health, making it even harder to seek help for physical health issues.

I find seeing a new doctor a very stressful experience now, especially if they already have my patient notes, because I have to prepare to not be listened to, talked down to, or entirely dismissed. (W, 26–35)

#### 3.4.1. People with MHSUC who need controlled drugs or pain management are particularly untrustworthy

Within this theme, the experience of being untrustworthy was intensified for people with addiction or taking controlled medication. Mistrust was most evident in relation to pain management and pain medication, and particularly affected people with addiction or perceived to be at risk of addiction, who could be labeled as drug seeking. They found it difficult to access pain relief and even routine investigation or treatment for symptoms.

Referred to mental health labeled as an attention/drug seeker. I had heart failure. (W, 55+)

The experience of mistrust extended beyond people with a history of addiction. People with mental health conditions but no history of addiction still felt they risked being labeled as a ‘drug seeker’ if they presented with pain. The consequence of this mistrust was that some people did not receive pain relief and others did not even ask for it, due to lack of confidence and a mutual lack of trust in clinicians and the health system.

I’m on a controlled drug for ADHD and feel that often I am treated as a criminal and a drug seeker both by my GP service and my regular pharmacy. Makes me loath to disclose any issue with pain or my anxiety as I know they will judge it as drug seeking behavior. (W, 36–45)

### 3.5. Mental and physical healthcare are competing priorities

People with MHSUC experienced two apparently conflicting assumptions related to the intersection between mental and physical healthcare, depending on the context and the focus of the clinician, which could be at odds with what the patient wanted to focus on.

#### 3.5.1. Mental healthcare takes the focus over physical health

The first assumption was that mental health issues must be attended to first before any physical health concerns could be addressed. The MHSUC was the primary or sole focus, even when people presented only with physical health concerns. Sometimes this was to the extent that clinicians appeared to be unable to deal with physical health conditions when there was co-existing MHSUC.

I went to seek help for a sore ankle, the Dr replied with a “tell me about your bipolar disorder.” Turns out I had a torn ligament, diagnosed by someone else. My treatment was delayed and I felt humiliated. (W, 36–45)

Respondents wanted to be seen as people, not as “mental health cases.” They wanted their physical symptoms to be treated as important and to be addressed fully and actively. They wanted physical causes to be ruled out first, rather than the MHSUC to dominate the consultation.

My mental health becomes a significant distraction and delays getting actual treatment for the issue I was there for. I wanted to know about my baby, but the conversation always went back to my mental health. I was stressed because I wanted to enjoy and understand my pregnancy, but no-one talked to me about my baby. I thought that’s what I was there for. (W, 36–45)

#### 3.5.2. Physical healthcare takes the focus over mental health

The second assumption pertaining to the relationship between physical and mental healthcare was that a person’s MHSUC had no impact on their physical health and that mental health concerns could be left to some other clinician or service. This appears to contradict the assumption that mental health takes the focus but demonstrates how mutually exclusive beliefs can exist within the same system.

Most descriptions of this assumption related to physical health services not understanding or accounting for stress and anxiety related to procedures, treatment or health settings or MHSUC being overlooked when it was relevant to physical healthcare.

I have Type 1 diabetes and am an outpatient at the diabetes clinic. Mental health is not addressed as a component of diabetes care, but in my experience, there is a lot of connection between my diabetes management and mental health. (M, 36–45)

Some of the tension between whether mental health or physical health was prioritized related to a lack of clarity over who was responsible for each domain of health. Holistic care, where physical and health issues were both treated as important and the interdependencies between them were recognized, was an ideal that respondents felt was rarely achieved.

The focus with some services is only on one thing – they do not take a holistic approach to wellbeing. If I am there for a physical issue then my mental health is not discussed and vice versa. (W, 26–35)

Short appointment times in primary care meant that some respondents felt that mental, addiction and physical health concerns could not both be adequately addressed in a single consult – this may have been dependent on service attributes of the practice and clinician. Although mental health was often assumed to be the cause of physical symptoms, the impact of physical health on mental health was seldom raised by clinicians.

## 4. Discussion

Respondents in this study provided many examples of how the demeanor and responses of clinicians would change or differ depending on their awareness of the respondent’s MHSUC, and the ways in which their MHSUC led to diagnostic and treatment overshadowing, contributing to delayed treatment and prolonged suffering.

Although research into the prevalence and impact of overshadowing is lacking (32), previous studies have documented adverse outcomes from diagnostic and treatment overshadowing (7, 10, 12). For example, in a qualitative study of clinicians working in the ED, clinicians recalled two patients with MHSUC who died and five who experienced irreversible long term damage due to delayed investigation or treatment (10). From the clinicians’ perspective, factors that contributed to overshadowing included difficulties in taking a detailed history, frequent attendances for unexplained symptoms and patients refusing to consent to an examination, procedure or treatment (10, 21). Poor interpersonal skills and lack of collaborative care from health providers were additional contributing factors (7, 21).

These studies convey how features of the patient and how they present contribute to overshadowing, explicitly or implicitly focusing the blame on the person. Treating patients differently because of any characteristic is discriminatory behavior, whether due to an individual clinician’s prejudice (33) or a system failure to accommodate the needs of patients (25, 28, 32, 34). By taking the perspective of the patient, we sought to focus on the clinicians’ actions, in order to shift responsibility onto services and systems to provide non-discriminatory care.

### 4.1. Addressing the psychosomatic assumption

Many respondents in this study reported that physical symptoms were attributed to their MHSUC almost by default, with no apparent consideration of a somatic cause. Without access to patient notes and records, we were unable to verify whether any or some appropriate investigations or examinations were conducted, but in many cases, the stories spoke for themselves – physical health conditions, including broken bones and infections, were missed.

This indicates not only discrimination but a failure in the duty of care, as presenting symptom(s) in all patients should be thoroughly investigated, regardless of previous history, which may include interviewing family or other contacts to gather additional information (10).

The assumption that MHSUC can cause physical symptoms may be true, as medically unexplained symptoms are extremely common, particularly in primary care (35). However, even in these situations, this does not mean that symptoms are not real and does not excuse a clinican from investigating for an organic cause and offering treatment or a referral if this is outside their expertise (36, 37). In the face of unexplained symptoms, clinicians are encouraged to reflect on the number of medical conditions throughout history that have been considered “psychosomatic” but subsequently found to have a biological basis (38).

### 4.2. Addressing stereotypes

Addressing the stereotype that people with MHSUC are untrustworthy involves more than one-off education sessions or increased awareness. From research on interventions to address unconscious bias, some strategies show promise (including exposure to counter-stereotypical examples, identifying with the outgroup and emphasizing the recovery from MHSUC) but positive effects may wane with time due to ongoing exposure to bias entrenched in society and discriminatory workplace cultures (13, 39). Interventions may need to be organization-wide and repeated, with ongoing assessment, reflection and deliberate practice (40, 41) using objective monitoring methods (e.g., internal audits against best practice standards, comparing treatment plans for people with and without MHSUC).

It is not only patients with MHSUC who are disbelieved and mistrusted. People reporting persisting symptoms after COVID infection were initially discounted, as “unreliable informants of their own illness experiences.” (42) People with MHSUC also experience discrimination in health care services due to belonging to other stigmatized groups, including female, ethnic minorities, sexual minorities and gender diverse people, which can worsen physical and mental health (43–46).

Not believing or taking a patient’s symptoms seriously is the antithesis of patient-centered care, a critical dimension of high-quality healthcare (40, 47, 48). A revised commitment to patient-centered care, with an explicit focus on respect, partnership, listening to and developing a trusted relationship with the patient, may be needed to improve quality of care for people with MHSUC.

### 4.3. Addressing mind–body dualism

The conflicting experiences of mental health taking precedence over physical health in some consultations and vice versa in others underscores the artificial and stigmatizing separation of mind and body within health systems (49, 50). Better integration between mental health, substance use and physical health services in order to improve outcomes for people with MHSUC is an ongoing challenge but a priority for health systems (51, 52). Characteristics of successful integration models include case management, care co-ordination and joint assessment/planning, shared information systems, co-location, clear accountabilities and strong leadership (51, 53–56). Physical health services, particularly primary care services which are often at the forefront of assessment, treatment and referral of people with MHSUC, need to have the requisite mental health and addiction training and skills (57, 58). Conversely, mental health and addiction services need to be aware of physical health risks associated with MHSUC and treatment and ensure that physical health needs are addressed (59).

### 4.4. Strengths and limitations

One limitation of this research was a relatively small sample size, but responses to the open-ended questions were high (71% of survey respondents gave textual answers), including some extensive narratives. However, we were unable to clarify ambiguous responses since the data were collected anonymously online. In this paper we focused on the worst experiences of physical healthcare for people with MHSUC. Respondents also recognized *non-*discriminatory behavior and provided exemplars of when they were treated well, which will be published separately. Responses to closed questions about quality and experience of healthcare services are published elsewhere (60). From this paper, 10% of respondents reported experiencing discrimination due to MHSUC always or most of the time, and this was significantly more prevalent in people with severe mental illness (schizophrenia or bipolar disorder), those with four or more diagnosis and LGBQA+ individuals. In addition, 20% of respondents reported diagnostic overshadowing always or most of the time, and this was experienced more often by Māori, people with severe mental illness or addiction and those with four or more diagnoses (60).

The underlying assumptions we have described are based on reports from respondents, and their interpretations of the clinician’s attitudes and behavior. We are unable to ascertain the beliefs and assumptions from the clinician’s perspective but regard the perceptions of respondents to be more valuable than clinician’s self-report in this context. Unconscious and social desirability biases are likely to lead to under-recognition and under-reporting of clinician bias against people with MHSUC. Taking the patient perspective, we are unable to disentangle whether unfair treatment is solely due to MHSUC or caused or compounded by other biases, such as racism, sexism or homophobia. More research should be done to examine the interactive impacts of belonging to more than one stigmatized group.

We were unable to quantify the impact or relative importance of assumptions that lead to discrimination against people with MHSUC. However, it is well established that people with MHSUC have worse outcomes from physical health conditions, including premature mortality (5). Further research is needed to quantify the extent of overshadowing and its causative factors and develop effective interventions to reduce it. One Australian survey found that 11% of people with MHSUC had experienced discrimination by a clinician in the previous 12 months (27).

The underlying assumptions identified in this research are consistent with findings from other studies on bias against people with MHSUC in clinicians (32), suggesting that these are not unique to NZ. However, in countries with different healthcare structures, particularly with higher levels of mental and physical healthcare integration, the competing priorities of mental and physical health may be less acute.

Respondents were self-selecting, recruited through social media and health service connections, hence may not reflect the experiences of people who are not engaged with health services or able to participate in online research. On the other hand, groups who might be more likely to experience other forms of discrimination such as women and people from the rainbow community were over-represented in our sample. However, although we would expect that more marginalized individuals would experience higher levels of discrimination, the stereotypes and underlying assumptions are likely to be similar.

## 5. Conclusion

Experiences of overshadowing in people with MHSUC are experiences of discrimination from individual clinicians, which may be exacerbated by personal and system factors, but are inherently healthcare quality issues. Interventions to change the way we support and manage the physical health of people with MHSUC are urgently needed.

## Data availability statement

The datasets presented in this article are not readily available because due to the nature of the research, respondents of this study did not agree for their data to be shared publicly, so supporting data are not available. Requests to access the datasets should be directed to ruth.cunningham@otago.ac.nz.

## Ethics statement

The studies involving humans were approved by Southern Health and Disability Ethics Committee. The studies were conducted in accordance with the local legislation and institutional requirements. Written informed consent for participation was not required from the participants or the participants’ legal guardians/next of kin because this was an online survey. Detailed information about the survey and contact details for more information was provided at the start of the survey. Informed consent was assumed when participants chose to engage with and complete the survey.

## Author contributions

RC: Conceptualization, Funding acquisition, Methodology, Project administration, Writing – review & editing. FI: Formal analysis, Methodology, Writing – original draft, Writing – review & editing. TH: Methodology, Writing – review & editing. SE-P: Methodology, Writing – review & editing. CL: Methodology, Writing – review & editing. HL: Conceptualization, Funding acquisition, Methodology, Writing – review & editing. DP: Conceptualization, Formal analysis, Funding acquisition, Methodology, Writing – review & editing.
